# Frequency and Risk Indicators of Tooth Decay among Pregnant Women in France: A Cross-Sectional Analysis

**DOI:** 10.1371/journal.pone.0033296

**Published:** 2012-05-07

**Authors:** Jean-Noel Vergnes, Monique Kaminski, Nathalie Lelong, Anne-Marie Musset, Michel Sixou, Cathy Nabet, for the EPIPAP group

**Affiliations:** 1 INSERM UMRS 953, Epidemiological Research Unit on Perinatal Health and Women’s and Children’s Health, Villejuif, France; 2 Department of Epidemiology, Faculty of Dentistry, Paul Sabatier University, Toulouse, France; 3 UMPC UnivP06, UMRS 953, Paris, France; 4 Faculty of Dentistry, Louis Pasteur University, Strasbourg, France; 5 Faculty of Dentistry, Paris Descartes University, Paris, France; 6 Charles Foix Hospital, Ivry/Seine, France; University of Southampton, United Kingdom

## Abstract

**Introduction:**

Little is known on the prevalence of tooth decay among pregnant women. Better knowledge of tooth decay risk indicators during pregnancy could help to develop follow-up protocols for women at risk, along with better prevention strategies. The aim of this study was to assess the frequency of tooth decay and the number of decayed teeth per woman in a large sample of pregnant women in France, and to study associated risk indicators.

**Methods:**

A secondary cross-sectional analysis of data from a French multicentre case-control study was performed. The sample was composed of 1094 at-term women of six maternity units. A dental examination was carried out within 2 to 4 days post-partum. Socio-demographic and behavioural characteristics were obtained through a standardised interview with the women. Medical characteristics were obtained from the women’s medical records. Risk indicators associated with tooth decay were identified using a negative binomial hurdle model.

**Results:**

51.6% of the women had tooth decay. The mean number of decayed teeth among women having at least one was 3.1 (s.d. = 2.8). Having tooth decay was statistically associated with lower age (aOR = 1.58, 95%CI [1.03,2.45]), lower educational level (aOR = 1.53, 95%CI [1.06,2.23]) and dental plaque (aOR = 1.75, 95%CI [1.27,2.41]). The number of decayed teeth was associated with the same risk indicators and with non-French nationality and inadequate prenatal care.

**Discussion:**

The frequency of tooth decay and the number of decayed teeth among pregnant women were high. Oral health promotion programmes must continue to inform women and care providers about the importance of dental care before, during and after pregnancy. Future research should also assess the effectiveness of public policies related to oral health in target populations of pregnant women facing challenging social or economic situations.

## Introduction

Tooth decay is a widespread, infectious disease classically related to the interplay of biological, behavioural and socio-economic influences. It affects about 40–50% of adults in industrialised countries [1], [2]. It has been hypothesised that pregnancy could increase the risk of caries initiation or progression, by changes in saliva composition [3], repeated gastric reflux or less effective oral health care [4]. However, given the relatively short time frame of pregnancy and the kinetics of dental caries progression [5], [6], it is unlikely that tooth decay will develop from initial carious lesion to major tooth damage within this period. Indeed, pregnancy in itself has never been clearly associated with an increased incidence of dental caries. Nevertheless, tooth decay is worth studying during pregnancy because the disease has potentially more critical consequences during this particular period. Tooth decay often leads to painful and stressful situations, with negative effects on the quality of life of pregnant women [7]. A recent study involving 504 pregnant women showed a 39% frequency of oral pain during pregnancy, predominantly caused by dental problems [7]. In this study, oral pain affected the subject’s normal activities much more than headaches and only a little less than back or pelvic pain. Unlike common pregnancy-related causes of pain and stress, tooth decay and subsequent development of dental pain could easily be avoided in most cases, because dental caries is a preventable disease. Self-medication and inappropriate use of analgesic medicines during pregnancy could also put the infant’s health at risk [8]. In addition, treatment of acute dental pain in emergency situations during pregnancy is delicate for the dental professional, with contraindications and the necessity for multiple precautionary measures [9].

**Table 1:** Frequency of tooth decay and number of decayed teeth, according to women’s characteristics.

	n^a^	%^a^	n^b^	%^b^	p^c^	OR^d^	95% CI^e^	Women with tooth decay	
								Mean^f^ (s.d.^g^)	p^h^
**Total**	1094	100	565	51.6				3.1 (2.8)	
**Age**	1094		565		<0.01				0.24
18–24	148	13.5	91	61.5		2.54	1.61–3.99	3.8 (2.9)	
25–29	331	30.3	180	54.4		1.77	1.22–2.55	3.0 (2.8)	
30–34	375	34.3	186	49.6		1.21	0.85–1.74	2.8 (2.5)	
≥35	240	21.9	108	45.0		1.00		3.5 (3.2)	
**Nationality**	1091		564		<0.01				<0.01
French	892	81.8	433	48.5		1.00		2.8 (2.6)	
Non-French	199	18.2	131	65.8		1.74	1.23–2.47	4.2 (3.3)	
**Marital status**	1094		565		0.41				0.14
Married	627	57.3	319	50.8		1.00		3.1 (2.6)	
Unmarried couple	395	36.1	204	51.6		1.09	0.83–1.44	3.1 (3.1)	
Living alone	72	6.6	42	58.3		1.41	0.83–2.39	4.0 (3.4)	
**Educational level**	1093		564		<0.01				<0.01
University	669	61.2	319	47.7		1.00		2.8 (2.4)	
Sixth form	192	17.6	106	55.2		1.53	1.08–2.17	3.4 (2.9)	
Compulsory education only	232	21.2	139	59.9		2.09	1.51–2.91	3.7 (3.5)	
**Employment during pregnancy**	1092		563		<0.01				<0.01
Yes	764	70.0	364	47.6		1.00		2.9 (2.6)	
No	328	30.0	199	60.7		1.82	1.37–2.41	3.7 (3.2)	
**Smoking status**	1091		562		0.04				0.74
Non-smoker	843	77.3	420	49.8		1.00		3.1 (2.6)	
Stopped smoking during pregnancy	141	12.9	79	56.0		1.19	0.80–1.75	3.5 (3.4)	
Smoking during pregnancy	107	9.8	63	58.9		1.73	1.11–2.67	2.9 (2.4)	
**Parity**	1093		564		0.78				0.52
Primiparous	569	52.1	295	51.8		1.04	0.80–1.34	3.1 (2.7)	
Multiparous	524	47.1	269	51.3		1.00		3.2 (3.0)	
**Adequate prenatal care**	1091		563		0.60				<0.01
Yes	974	89.3	499	51.2		1.00		3.1 (2.6)	
No	117	10.7	64	54.7		1.14	0.70–1.85	4.0 (4.0)	
**BMI before pregnancy**	1082		557		0.13				0.66
<18.5	9.2	8.5	53	57.6		1.44	0.90–2.30	3.7 (3.4)	
[18.5–25[	761	70.3	383	50.3		1.00		3.0 (2.7)	
[25–30[	151	14.0	75	49.7		1.10	0.76–1.59	3.2 (2.9)	
> = 30	78	7.2	46	59.0		1.65	1.00–2.72	3.6 (3.0)	
**High quantity of plaque**	1094		565		<0.01				<0.01
No	354	32.4	135	38.1		1.00		2.0 (1.2)	
Yes	740	67.6	430	58.1		1.92	1.42–2.61	3.5 (3.1)	
**High quantity of calculus**	1094		565		<0.01				<0.01
No	843	77.1	412	48.9		1.00		2.9 (2.6)	
Yes	251	22.9	153	61.0		1.67	1.18–2.36	3.7 (3.2)	
**Last visit to the dentist**	1092		564		<0.01				<0.01
Less than one year before pregnancy	813	74.4	394	48.5		1.00		2.9 (2.6)	
More than one year before pregnancy	279	25.6	170	60.9		1.60	1.18–2.16	3.6 (3.1)	

a Number and percentage of women in each class of the variables.

b Number and percentage of women with tooth decay in each class of the variables.

c Wald χ^2^ test adjusted for examiner.

d Odds Ratio adjusted for examiner.

e 95% Confidence Interval.

f Mean number of decayed teeth in each class, among women with tooth decay.

g Standard deviation.

h General linear models (F-test) adjusted for examiner.

The few studies assessing the frequency of tooth decay during pregnancy report values between 47% and 69% [7], [10], [11]. These recent studies were conducted on relatively small populations in Pakistan, Brazil and Hungary. To our knowledge, there is no previous study reporting data on the frequency of tooth decay among pregnant women in France. Neither have we found any international study investigating the risk indicators specifically related to decayed teeth in pregnant women. Better knowledge of the prevalence of tooth decay and associated risk indicators during pregnancy would help to develop follow-up protocols for women at risk, along with better prevention strategies.

**Figure 1:**
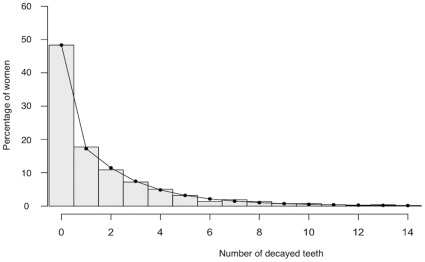
Distribution of number of decayed teeth per woman. The bars represent the values observed in the sample of 1094 women. The curve represents the values predicted by the Hurdle model.

The objectives of this study were to assess the frequency of tooth decay and the number of decayed teeth in a large sample of pregnant women in France, and to study associated risk indicators.

**Table 2:** Risk indicators for tooth decay: results from the multivariate analysis^a^.

Variables	Logistic portion^b^			Negative binomial portion^c^		
	aOR^d^	95% CI^e^	p-value	Adjusted exp(β)^f^	95% CI	p-value
**Age (years)**						
18–24	1.58	[1.03,2.45]	0.03*	1.37	[1.06,1.78]	0.01*
25–29	1.59	[1.16,2.17]	<0.01*	1.05	[0.85,1.29]	0.62
≥30	1.00			1.00		
**Nationality**						
French	1.00			1.00		
Non-French	1.48	[1.00,2.19]	0.05	1.30	[1.04,1.62]	0.02*
**Educational level**						
University	1.00			1.00		
Sixth form	1.15	[0.78,1.67]	0.45	1.19	[0.92,1.54]	0.17
Compulsory education only	1.53	[1.06,2.23]	0.02*	1.40	[1.09,1.79]	<0.01*
**Employment during pregnancy**						
Yes	1.00			1.00		
No	1.35	[0.97,1.86]	0.07	1.15	[0.93,1.41]	0.18
**Smoking**						
Non-smoker	1.00			1.00		
Stopped smoking during pregnancy	1.11	[0.73,1.68]	0.60	1.19	[0.87,1.47]	0.18
Smoker during pregnancy	1.49	[0.93,2.39]	0.09	0.83	[0.51,1.14]	0.26
**Adequate prenatal care**						
Yes	1.00			1.00		
No	0.80	[0.47,1.37]	0.41	1.46	[1.08,1.99]	0.01*
**High quantity of plaque**						
Yes	1.75	[1.27,2.41]	<0.01*	1.82	[1.38,2.42]	<0.01*
No	1.00			1.00		
**High quantity of calculus**						
Yes	1.41	[0.97,2.05]	0.06	1.23	[1.00,1.53]	0.05
No	1.00			1.00		
**Last visit to the dentist**						
Less than one year before pregnancy	1.00			1.00		
More than one year before pregnancy	1.31	[0.94,1.80]	0.09	1.18	[0.97,1.44]	0.08

a Hurdle model, adjusted for all the variables in the table plus examiner to account for inter-examiner variability.

b Models the probability of the women having tooth decay.

c Models the number of decayed teeth among the women having at least one.

d Adjusted odds ratio.

e 95% Confidence Interval.

f exp(β) can be interpreted as follows: while holding all other variables constant in the model, among women having tooth decay, a woman in a given class has on average exp(β) more decayed teeth than a woman in the reference class. For example, among women having tooth decay, a woman aged 18–24 years has on average 1.37 more decayed teeth (or 37% more decayed teeth) than a woman aged 30 or more (reference class for age).

* Significant at α = 5%.

## Methods

### Ethics Statement

The study was approved by the French data protection authority, and all the women included gave their written informed consent.

### Study Sample

The study sample was made up of 1094 women who had given birth to a singleton live-born infant at term (≥37 weeks), randomly selected between 2003 and 2006 in 6 maternity units of 3 French regions (Ile-de-France, Midi-Pyrénées and Alsace). This sample formed the control group of the EPIPAP study, a multicentre case-control study which primarily aimed to analyse the association between periodontitis and preterm birth, according to the main causes of preterm birth [12]. Oral health comparisons between cases (women with delivery term at <37 weeks’ gestation) and controls (women with delivery term at ≥37 weeks’ gestation) have been published elsewhere [12], [13]. Only the control group of the EPIPAP case-control study was used in this cross-sectional analysis. Non-inclusion criteria were: age under 18, not understanding the French language, HIV infection, unbalanced diabetes or any medical condition that required antibiotic prophylaxis for dental examination and periodontal probing, fewer than 6 teeth, and infant born with a severe congenital malformation.

### Data

Examinations were performed within 2 to 4 days post-partum, in the post-delivery wards of the maternity units. It was considered that tooth decay observed within 4 days post-partum was already present during pregnancy. Women were examined in a sitting position. The eleven dentists in charge of the oral examinations performed intra-oral screening to ascertain the amount of plaque, calculus and gingival inflammation, clinical attachment level, periodontal pocket depth, bleeding on probing, and presence of tooth decay and fillings. Examiners were given instructions to assess carious lesions according to the World Health Organisation (WHO) diagnosis criteria [14]. The presence of carious lesions was recorded at the surface level of the teeth using sterile dental mirrors and explorers. A carious lesion was defined as a cavity that appeared as a darkened hole with irregular breakdown of the enamel surface. Stain and pigmentation alone were not considered as carious lesions neither were white spot lesions, nor apparent tooth wear or erosion. Four surfaces were examined and coded for incisors and canines, and five surfaces for premolars and molars. Third molars were excluded from the assessment and radiographs were not taken. A decayed surface was recorded when at least one carious lesion could be observed on a surface, including carious lesion contiguous with the margin of a filling. Analyses were performed at tooth level. A decayed tooth was a tooth with at least one decayed surface. A woman was considered as having tooth decay if at least one of her teeth was decayed.

Amounts of plaque and calculus were measured at four sites per tooth, using the Silness-Löe plaque index [15] and the Greene and Vermillion calculus index [16]. A woman was classified as having a high quantity of plaque if the examiner reported at least one site with visible plaque on at least one tooth. Similarly, a woman was classified as having a high quantity of calculus if the examiner reported at least one site with calculus covering more than one third of the exposed tooth surface of at least one tooth. Adequacy of dental attendance was assessed through the variable ‘time since last visit to dentist’ (consistent with clinical guidelines for patients aged 18 years and older: less than one year before pregnancy; or not consistent with clinical guidelines: more than one year before pregnancy or never) [17].

Socio-demographic and behavioural characteristics were obtained through a standardised interview of the women after the dental examination. Medical characteristics were obtained from the women’s medical records. All examiners were blinded to the medical and socio-demographic data. Socio-demographic characteristics included age (18–24,25–29,30–34 and ≥35 years), nationality (French or not), marital status (married, unmarried couple, living alone), educational level (primary and secondary compulsory education, sixth form, university), and employment during pregnancy (yes/no). Behavioural characteristics were smoking status during pregnancy (non smoker before pregnancy, stopped smoking during pregnancy, smoker during pregnancy), and adequacy of prenatal care, assessed by the number of prenatal visits according to gestational age at delivery with reference to French regulations. Medical characteristics were the Body Mass Index (BMI) and the parity of the mother (primiparous vs multiparous). BMI was calculated by dividing weight (in kilograms) by the square of the height (in metres), and assessed using self-reported values of height and weight before pregnancy. BMI values were classified into four categories: less than 18.5, 18.5 to 24.9, 25 to 29.9 and more or equal to 30.

### Statistical Analysis

Descriptive analysis of the sample was performed using relative percentages for each class of categorical variables. The proportion of women with tooth decay was also presented according to the women’s characteristics. Bivariate analyses were conducted to identify women’s characteristics associated with tooth decay and number of decayed teeth. Risk indicators associated with tooth decay were identified using the Wald Chi-2 test adjusted for examiner, and odds ratios (ORs) and their 95% confidence intervals (95% CI) were calculated. In order to avoid losing quantitative information, the number of decayed teeth per woman was also calculated, hypothesising that the risk of caries-related problems during pregnancy increased with the number of decayed teeth. Among women having at least one decayed tooth, risk indicators associated with the number of decayed teeth were identified using general linear models (F-test) adjusted for examiner.

Risk indicators associated with tooth decay or the number of decayed teeth were analysed together using a hurdle model, a two-component regression model for count outcomes [18]. Hurdle models are appropriate for modelling count data with excess zeros [19], which is the case for the number of decayed teeth per person in adult populations of contemporary industrialised countries [20]. This model first uses logistic regression to predict the probability of the woman having any decayed teeth, then it calculates the conditional expectation of the number of decayed teeth for the subsample of only the women who have at least one. The count part of the model is a truncated negative binomial regression (with log link) [18]. All women’s risk indicators significantly associated with tooth decay and/or the number of decayed teeth in the bivariate analysis were included in the model. Ordinal variables with more than two classes were dummy coded for the regression procedures. The multivariate analysis was also adjusted for examiner to take the inter-examiner variability into account. The adequacy of the model was assessed using the Wald Chi-2 test, and predicted values of number of decayed teeth were calculated.

The significance level was set at p<0.05. Statistical analyses were performed using SAS software version 9, and R software version 2.7.1, with the additional *pscl* package version 1.02 (hurdle() function).

## Results

18.2% of the women were not French, 6.6% were living alone, 21.2% had a low educational level, and 30.0% were not employed during pregnancy (Table 1). On the whole, 51.6% of the women had tooth decay. Among women who had tooth decay, the mean number of decayed teeth was 3.1 (sd = 2.8). Both the frequency of tooth decay and the mean number of decayed teeth were significantly associated with non-French nationality, lower educational level, unemployment during pregnancy, high quantity of plaque, high quantity of calculus and time since last visit to the dentist. The frequency of women with tooth decay was higher among lower age groups and among smokers during pregnancy (Table 1). The mean number of decayed teeth was also higher when prenatal care was inadequate (Table 1). In contrast, neither presence of tooth decay nor number of decayed teeth were associated with marital status, parity, or BMI before pregnancy.

Figure 1 represents the observed distribution of the number of decayed teeth per woman. The distribution is skewed to the right, with 48.4% of ‘caries-free’ women. The curve shows the predicted distribution of the number of decayed teeth per woman obtained from the multivariate analysis and indicates a good fit of the model to the data (Figure 1). The hurdle model was significant (Wald Chi-2 test, p<0.0001), meaning that at least one of the regression coefficients was not equal to zero.

Table 2 shows the results of the multivariate analysis (Hurdle model) between women’s characteristics and both existence of tooth decay and number of decayed teeth. In the logistic portion of the hurdle model, lower age groups, low educational level and high quantity of plaque were independently associated with a higher risk of tooth decay. Non-French nationality was borderline significant. In the negative binomial portion, the number of decayed teeth was associated with the same risk factors and with non-French nationality and inadequate prenatal care. Comparatively to women aged 30 years and more, women in the 18–24 age group presented 1.37 more decayed teeth on average, i.e. 37% more decayed teeth. Non-French women had on average 30% more decayed teeth than French women. Women with an educational level of primary or compulsory secondary school had on average 40% more decayed teeth than more highly educated women, and women with inadequate prenatal care during pregnancy presented on average 46% more decayed teeth than women with adequate prenatal care.

## Discussion

We showed that more than 50% of the pregnant women had tooth decay. Having tooth decay was associated with lower age and lower educational level. The number of decayed teeth was associated with the same risk indicators, and with non-French nationality and inadequate prenatal care.

This study was limited to six maternity units, so the frequency of tooth decay in the overall population of pregnant women in France cannot be inferred from the present data. However, women included in this study had characteristics similar to those of women from the French 2003 National Perinatal Survey [21]. A limitation of this study was the use of data from a case-control study not originally designed to address questions of frequency of tooth decay. We chose to account for the sampling design of the original study by performing a cross-sectional analysis of the controls only [22]. A naive analysis disregarding the sampling strategy that gave rise to the data would be prone to bias, through an over-representation of cases. Dental examiners were standardised and were given clear instructions prior to the observation period to assess dental caries according to the WHO diagnosis criteria [14]. However, calculation of inter-examiner reliability was not performed. We thus chose to account for possible residual inter-examiner variability by adjusting for examiner in the statistical models [13]. Finally, an objective of this work was to study the risk indicators associated with tooth decay during pregnancy. As stated by Burt [23], a risk indicator may be a putative risk factor, but the cross-sectional data upon which it is based is weaker than the results of longitudinal studies. Another limitation of this study is that we did not explore some variables that could be considered as important risk indicators for tooth decay, such as dietary habits or dental hygiene habits. The primary EPIPAP study was designed to analyse the association between maternal periodontitis and preterm birth according to causes of preterm birth, so dietary habits were not collected. Given the putative overestimation of self-reported oral hygiene practices, we considered the presence of plaque and calculus as more direct risk indicators for tooth decay.

The frequency of dental caries in our sample was similar to the prevalence observed in the general adult population of the same age [2], [20], [24], although dental studies among the general adult population remain rare. Our results (51% of women with tooth decay) are in agreement with the frequency of dental caries among pregnant or post-partum women reported in previous studies from other countries. In a Pakistani cohort study of 1152 pregnant women (mean age 26.5 years), nearly 47% of the women had at least one decayed tooth [10]. The prevalence of tooth decay was 61% in a sample of 504 low-income Brazilian pregnant women (mean age 24 years) [7]. A Hungarian study found that 69% of postpartum mothers (mean age 27.5 years) required one or more restorations [11]. In all these studies, as well as ours, the conditions of dental examination might have led to an underestimation of both frequency of tooth decay and number of decayed teeth.

Lower age, non-French nationality and low educational level were related to both frequency of tooth decay and number of decayed teeth. We found that 18–24 year-old women were at higher risk for tooth decay than the older ones, independently of the amount of dental plaque and adequacy of dental attendance. Lower age as a risk indicator for tooth decay has already been described in France [24]. In 2004, a study involving about 600 000 adults showed that the highest proportion of subjects with at least one untreated carious lesion was among the 20–24 age group [24]. Although not explored in this study, younger women (aged 18–24 years) could be at higher risk of dental caries because snacking has been shown to be common in this age group [25]. Another explanation from the literature could be that 18–24 year-old adults are less likely to regularly visit a dental professional than other age groups [26]. Even if we adjusted for adequacy of dental attendance, it is likely that this binary variable would not fully reflect the preventive behaviour of the included women.

Non-French nationality was found to be associated with higher risk of having tooth decay. In France, the current nationality of the mother is a variable widely used in epidemiological studies [27], [28], [29] as ethnic category does not cover the notion of migration. It was important to take the woman’s nationality into account in the multivariate analysis because it has been shown that a lack of regular medical care stems from social obstacles, especially in foreign women [30]. For example, it has been shown that immigrant women are at risk of poor pregnancy outcomes [31], and that immigrant status is a significant caries predictor in children living in a deprived area [32]. Poor availability of translations and of culturally competent services may constitute an obstacle to a contributive medical visit [31]. Further studies are needed to elucidate the obstacles to optimal management of these women in the French model of healthcare organisation, which is based on the principle of universal access to care [31].

A lower educational level was also found to be a significant risk factor for the frequency of tooth decay and the number of decayed teeth, which is consistent with previous studies showing that low educational level can be considered as a major risk factor for dental caries [33]. In the present study, women in the lower educational levels were more likely to declare insufficient dental attendance (data not shown). These data corroborate a French national study showing that subjects of lower educational levels were less likely to visit a dentist annually [34].

In conclusion, the frequency of tooth decay and the number of decayed teeth among pregnant women were high. Oral health promotion programmes need to inform pregnant women, prenatal care providers and oral health professionals about the particular importance of dental care before, during and after pregnancy. Future research should also assess the effectiveness of public policies related to oral health among some target groups of pregnant women facing challenging social or economic situations.
